# FolC2‐mediated folate metabolism contributes to suppression of inflammation by probiotic *Lactobacillus reuteri*

**DOI:** 10.1002/mbo3.371

**Published:** 2016-06-28

**Authors:** Carissa M. Thomas, Delphine M. A. Saulnier, Jennifer K. Spinler, Peera Hemarajata, Chunxu Gao, Sara E. Jones, Ashley Grimm, Miriam A. Balderas, Matthew D. Burstein, Christina Morra, Daniel Roeth, Markus Kalkum, James Versalovic

**Affiliations:** ^1^ Integrative Molecular and Biomedical Sciences (IMBS)Baylor College of MedicineOne Baylor PlazaHoustonTexas77030; ^2^ Department of Pathology & ImmunologyBaylor College of MedicineHoustonTexas; ^3^ Department of PathologyTexas Children's Hospital1102 Bates AveHoustonTexas77030; ^4^ Department of Molecular Virology and MicrobiologyBaylor College of MedicineHoustonTexas; ^5^ Structural and Computational Biology and Molecular Biophysics Graduate ProgramBaylor College of MedicineHoustonTexas; ^6^ Department of Molecular ImmunologyBeckman Research Institute of the City of Hope1500 E Duarte Rd.DuarteCalifornia91010; ^7^ Present address: Microbiome and Inflammation Start up LabDepartment of Gastrointestinal MicrobiologyGerman Institute of Human NutritionPotsdam‐RehbrückeGermany

**Keywords:** Colitis, folate, *folC2*, histamine, immunomodulation, *Lactobacillus reuteri*.

## Abstract

Bacterial‐derived compounds from the intestinal microbiome modulate host mucosal immunity. Identification and mechanistic studies of these compounds provide insights into host–microbial mutualism. Specific *Lactobacillus reuteri* strains suppress production of the proinflammatory cytokine, tumor necrosis factor (TNF), and are protective in a mouse model of colitis. Human‐derived *L. reuteri* strain ATCC PTA 6475 suppresses intestinal inflammation and produces 5,10‐methenyltetrahydrofolic acid polyglutamates. Insertional mutagenesis identified the bifunctional dihydrofolate synthase/folylpolyglutamate synthase type 2 (*folC2*) gene as essential for 5,10‐methenyltetrahydrofolic acid polyglutamate biosynthesis, as well as for suppression of TNF production by activated human monocytes, and for the anti‐inflammatory effect of *L. reuteri* 6475 in a trinitrobenzene sulfonic acid‐induced mouse model of acute colitis. In contrast, *folC* encodes the enzyme responsible for folate polyglutamylation but does not impact TNF suppression by *L. reuteri*. Comparative transcriptomics between wild‐type and mutant *L. reuteri* strains revealed additional genes involved in immunomodulation, including previously identified *hdc* genes involved in histidine to histamine conversion. The *folC2* mutant yielded diminished *hdc* gene cluster expression and diminished histamine production, suggesting a link between folate and histadine/histamine metabolism. The identification of genes and gene networks regulating production of bacterial‐derived immunoregulatory molecules may lead to improved anti‐inflammatory strategies for digestive diseases.

## Introduction

*Lactobacillus reuteri* is a vertebrate symbiont found in the gastrointestinal (GI) tract of a variety of mammalian species and considered indigenous to the human gut (Reuter 2001; Walter et al. 2011). Several *L. reuteri* strains are probiotics, “viable microorganisms that confer a health benefit to the host when administered in adequate amounts” (FAO/WHO, 2006). Selective deficiencies of intestinal lactobacilli have been described in patients with inflammatory bowel disease (IBD) (Giaffer et al. 1991; Vigsnaes et al. 2012; Zella et al. 2011), and oral or intrarectal supplementation with various probiotic *Lactobacillus* species effectively ameliorates intestinal inflammation in patients with pouchitis (Bibiloni et al. 2005; Gupta et al. 2000) and in rodent colitis models (Foligne et al. 2006; Holma et al. 2001; Moller et al. 2005; Pena et al. 2005; Schreiber et al. 2009; Satish Kumar et al. 2015; Liu et al. 2011; Peran et al. 2007). In vitro studies have demonstrated that lactobacilli possess species‐specific, potent immunosuppressive activities, such as modulation of murine dendritic cell‐induced differentiation of Th1 and Th2 cells (Christensen et al. 2002), increasing production of IL‐10 from dendritic cells and macrophages (Livingston et al. 2010; Bleau et al. 2010), inhibiting production of TNF from lipopolysaccharide (LPS)‐stimulated monocytes (Kim et al. 2008), and driving development of IL‐10‐producing regulatory T cells (Smits et al. 2005; Zhao et al. 2013). The immunosuppressive functions of probiotics, like *L. reuteri*, could be harnessed to make new therapeutics for chronic autoimmune or inflammatory disorders.

The reduction in biologically active, circulating TNF by neutralizing antibodies has been an effective treatment strategy for patients with IBD (Peyrin‐Biroulet 2010) and trinitrobenzene sulfonic acid (TNBS)‐challenged rats (Triantafillidis et al. 2005). Anti‐TNF strategies, however, are complicated by secondary deficiencies in antimycobacterial immunity and possible sensitization or development of antibodies to these therapies (Hoentjen and van Bodegraven 2009; Jauregui‐Amezaga et al. 2013; Ungar et al. 2014). Additionally, anti‐TNF therapies have been associated with reactivation of hepatitis B virus and a slightly increased risk of melanoma (Chebli et al. 2014). These side effects make anti‐TNF strategies less desirable as long‐term therapeutics. In contrast to systemic antibody‐based strategies, luminal bacteria in the intestine may be able to suppress inflammation and proinflammatory cytokine activities in a gut‐specific manner. Bacterial‐derived, cell‐free culture supernatants of human‐derived *L. reuteri* ATCC PTA 6475 (6475) and *L. reuteri* CRL1098 suppressed TNF production by primary monocyte‐derived macrophages from patients with Crohn's disease and activated myeloid cell lines (Lin et al. 2008; Pena et al. 2005) and activated peripheral blood mononuclear cells (Mechoud et al. 2012). *L. reuteri* 6475 biofilms were capable of suppressing TNF production by LPS‐activated monocytoid cells (Jones and Versalovic 2009). A combination of *L. reuteri* 6475 and *L. paracasei* reduced colonic TNF as well as intestinal inflammation in an IL‐10‐deficient, *Helicobacter hepaticus*‐induced IBD mouse model (Pena et al. 2005), and a mixture of four *L. reuteri* strains was protective in a dextran sodium sulfate (DSS)‐induced colitis rat model (Schreiber et al. 2009). These previously published results suggest that *L. reuteri* strains may be effective immunoregulatory probiotics, and autochthonous components of the gut microbiome may affect the biology of the mucosal immune system.

Probiotic supernatants and cell‐derived factors inhibit cytokine production and suppress inflammatory signaling in macrophages and other immune cells (Grangette et al. 2005; Thomas and Versalovic 2010), but a paucity of bacterial genes and products required for immunomodulation have been identified (Grangette et al. 2005; Yasuda et al. 2008). Recently the biogenic amine, histamine, was identified as a TNF‐inhibitory factor produced by *L. reuteri* 6475. Histamine is produced by the decarboxylation of l‐histidine, and *L. reuteri*‐mediated histamine production can be increased by histidine supplementation in the growth medium (Thomas et al. 2012). *L. reuteri* also synthesizes the essential B‐complex vitamin, folate, when a precursor para‐aminobenzoic acid (pABA), is provided in the medium (Spinler et al. 2014; Rossi et al. 2011; Santos et al. 2008). In selected microorganisms, folate may catalyze one‐carbon units into histidine, suggesting that folate may be involved in histidine biosynthesis (Broquist 1957). Crosstalk between these microbial metabolic pathways may result in the coregulation of histamine and folate biosynthesis. Additionally, in eukaryotic cells, it is known that oxidation of amino acids, including histidine, is linked to folate metabolism. Folate plays a key role in the reduction in NAD^+^ to NADH and NADP^+^ to NADPH in the oxidation–reduction reactions necessary for one‐carbon metabolism (Brosnan et al. 2015). Similar reactions may occur in prokaryotes, reinforcing the possible coregulation of histamine and folate biosynthesis in *L. reuteri*.

Greater understanding of bacterial immunomodulatory gene networks and mechanistic studies of immunomodulatory compounds should improve selection of effective probiotics for specific therapeutic applications. In this era of microbiome science, functional linkages between different microbial metabolic pathways may elucidate mechanisms of probiosis and immunoregulation by gut microbes. The goal of this study was to identify immunomodulatory genes and regulatory networks present in TNF‐inhibitory *L. reuteri* 6475. These studies demonstrated a novel role for the dihydrofolate synthase/folylpolyglutamate synthase gene type 2 (*folC2*) in TNF suppression and colitis attenuation, and demonstrate a potentially important link between folate metabolism and histamine production.

## Experimental Procedures

### Bacterial strains and culture conditions

All bacterial strains and plasmids used in this study are described in Table S2. *L. reuteri* strains ATCC PTA 6475, ATCC 6475 *folC2*::pORI28, ATCC 6475 *folC*::pORI28, and ATCC 55730, are referred to as strains 6475, 6475::*folC2*, 6475::*folC*, and 55730, respectively. *L. reuteri* strains were cultured for 24 h at 37°C in an anaerobic workstation (MACS MG‐500, Microbiology International, Frederick, MD) supplied with a mixture of 10% CO_2_, 10% H_2_, and 80% N_2_ for 16–18 h in de Man–Rogosa–Sharpe (MRS) medium (Difco, Franklin Lakes, NJ), and then inoculated into a defined medium, LDMIII (OD_600_ adjusted to 0.1), which has been described previously (Jones and Versalovic 2009). At stationary phase (24 h), the cells were pelleted (4000*g*, 10 min). Insertion mutants were cultured in the presence of 10 *μ*g/mL erythromycin.

### Construction *folC2* and *folC* insertion mutants (*L. reuteri* 6475::*folC2* and 6475::*folC*)

Bifunctional dihydrofolate synthase/folylpolyglutamyl synthase type 2 (*folC2*) and bifunctional dihydrofolate synthase/folylpolyglutamyl synthase (*folC*) genes were identified in the whole draft genome sequence of *L. reuteri* 6475 (GenBank NZ_ACGX02000001‐007; HMPREF0536_11260 and HMPREF0536_10555, respectively). Inactivation of these genes was achieved by site‐specific integration of plasmid pORI28 into the *L. reuteri* 6475 chromosome as described previously (Walter et al. 2005). Briefly, internal gene fragments were amplified by PCR (outlined in Table S2) and directionally cloned into pORI28. Site‐specific homologous recombination of target‐specific pORI28 vectors was performed as detailed by Walter et al. (2005). Site‐specific insertional mutagenesis was confirmed by dideoxy DNA sequencing.

### Tetrahydrofolic acid compound analysis by MALDI mass spectrometry

Cell pellets normalized by weight from wild‐type and mutant *L. reuteri* strains were washed with ice‐cold PBS and water. Folates were extracted by washing with 50% acetonitrile/0.1% v/v TFA. The cell suspension was centrifuged for 10 min, 4000*g* at 4°C. Samples were prepared with *α*‐cyano‐4‐hydroxy‐cinnamic acid as MALDI matrix, spotted onto a sample plate, dried, and analyzed on a prOTOF2000 MALDI mass spectrometer (PerkinElmer/Sciex, Boston, MA) or on a SimulTOF Combo 200 MALDI mass spectrometer (Virgin Instruments Marlborough, MA). For MS/MS fragmentation analysis, the same MALDI sample plate was subsequently transferred into a self‐built MALDI quadrupole ion trap that was based on a modified LCQ DECA iontrap (Thermo Waltham, MA) (Krutchinsky et al. 2001). Tetrahydrofolate ions were fragmented with a 4 Da selection window, 30% collision energy, a fixed ion trap injection time of 200 msec, and a 2–10‐hz laser repetition rate.

### Assessment of TNF inhibition by ELISA

Bacterial supernatants from a 24 h LDMIII culture were filter‐sterilized using polyvinylidene fluoride membrane filters (0.22 *μ*m pore size, Millipore, Bedford, MA) and size‐fractionated with Amicon Ultra‐15 centrifugal filter units using ultracel‐3 membrane (Millipore). The filtrate was speed vacuum‐dried and resuspended in RPMI medium. All supernatants were normalized by volume to OD_600_ = 1.5. Supernatants and cell pellet washes were tested for their ability to modulate TNF production. *In vitro* experiments were performed with THP‐1 cells (human monocytoid cell line, ATCC number TIB‐202, ATCC, Manassas, VA) maintained in RPMI (ATCC) and heat‐inactivated fetal bovine serum (Invitrogen, Carlsbad, CA) at 37°C, with 5% CO_2_. THP‐1 cells (5 × 10^4^ cells) were stimulated to produce TNF by the addition of 100 ng/mL Pam_3_Cys‐SKKKK x 3 HCl (EMC Microcollections, Tüebingen, Germany) as previously described (Pena et al. 2004). *L. reuteri* supernatant or cell pellet wash was added to the activated THP‐1 cells (5% v/v). Plates were incubated at 37°C and 5% CO_2_ for 3.5 h. THP‐1 cells were pelleted (3000*g*, 5 min, 4°C), and quantitative ELISAs were used to determine TNF quantities in THP‐1 cell supernatants according to the manufacturer's instructions (R&D Systems, Minneapolis, MN).

### TNF gene expression studies by qPCR

THP‐1 cells were treated with *L. reuteri* cell‐free supernatant and PCK as described above. RNA isolation was performed with the AllPrep DNA/RNA mini kit from Qiagen (Valencia, CA) according to manufacturer's instructions. RNA quantity and quality was assessed, and only RNA with RIN greater than or equal to 9 was used in the subsequent assays. Gene expression was analyzed using the RT^2^ Profiler PCR Array (innate and adaptive immune response) from Qiagen according to manufacturer's instructions. In brief, cDNA was prepared from purified RNA with the RT^2^ first strand kit. The cDNA was added to RT^2^ SYBR green mastermix, and aliquoted into the 96‐well RT^2^ Profiler PCR Array of interest. All PCR reactions were performed using the Stratagene Mx3005P PCR System. Cycling parameters were as follows: program 1; One cycle of 95°C for 10 min, program 2; 40 two‐step cycles of 95°C for 15 sec, 60°C for 1 min, program 3; hold at 4°C. Fluorescence was detected after the extension step in each cycle. The 2^−ΔΔCT^ method was used to calculate relative changes in gene expression.

### Transcriptomics comparisons of *L. reuteri* mutants

*L. reuteri* 6475, 6475::*folC2*, and 6475::*folC* were cultured in LDMIII to stationary phase (24 h). For expression analyses, three biological replicates were performed with dye‐swap experiments for each strain/mutant. Following mRNA isolation, cDNA synthesis, labeling, and hybridization were performed as previously described (Wall et al. 2007; Yang et al. 2005). Information regarding the microarray platforms is at the NCBI Gene Expression Omnibus (GEO; http://www.ncbi.nlm.nih.gov/geo/) under GEO platform GPL754. The complete set of microarray data for 6475::*folC2* (formerly known as 6475::*thfs*) and 6475::*folC* (formerly known as 6475::*thfs2*) can be found under the GEO series accession GSE32971 and GSE32972, respectively. Microarray data analysis was performed as previously described (Maguin et al. 1992) utilizing the array package in R2.12.1. The gene set of interest (GSI) was the list of genes potentially contributing to the immunomodulatory phenotype of strain 6475. The GSI was refined by analyzing subsequent layers of gene expression data. The GSEA v2.07 analysis was performed using the online analysis tool (http://www.broadinstitute.org/gsea/index.jsp). The 402 genes significantly up‐regulated in stationary phase of 6475 that were missing a homolog or not significantly up‐regulated in stationary phase of 55,730 were used as the input Gene Set. The ranked list of 6475::*folC2* expression pattern via fold change was used as the preranked input. A weighted enrichment statistic was calculated using 1000 permutations. The core enriched and down‐regulated 6475::*folC2* genes were further refined by removing genes significantly down‐regulated in 6475::*folC*. These potential immunoregulatory genes were sorted by a combined DEDS statistic which placed equal weight on 6475::*folC2* and 6475 stationary phase linear fold changes (Yang et al. 2005). DEDS represents the selected differential expression measures from the previous analyses as a multivariate point cloud with multiple dimensions associated with different input statistics. The scalar distance to the most extreme combination of these input statistics, following permutation, is the final metric by which genes are ranked and displayed here.

### Gene expression studies of the *L. reuteri hdc* cluster

*L. reuteri* 6475 and 6475::*folC2* were grown as described above in LDMIII or LDMIII + 4 mg/mL l‐histidine. At 16 h post‐inoculation, the cultures were harvested. RNA isolation and cDNA synthesis from total RNA were performed as previously described (Thomas et al. 2012). Expression of the *hdcA, hdcB, hdcP,* and *narI* genes was analyzed using quantitative real‐time PCR. All primers were designed using the Universal Probe Library Assay Design Center (Roche Applied Science, Indianapolis, IN) and are described in Table S2. The RNA polymerase *β*‐subunit (*rpoB*) gene was used as a reference gene. PCR reactions were set up using 2× FastStart Universal Probe Master (Rox) (Roche Applied Science) and the cDNA described above, with final concentrations of 200 nmol/L for each primer and 100 nmol/L for each probe. All PCR reactions were performed using the ViiA 7 Real‐Time PCR System (Life Technologies, Carlsbad, CA). Cycling parameters were as follows: program 1; One cycle of 25°C for 2 min, one cycle of 95°C for 10 min, program 2; 50 three‐step cycles of 95°C for 15 sec, 60°C for 1 min and 72°C for 1 min, program 3; hold at 4°C. Fluorescence was detected after the extension step in each cycle. The 2^−ΔΔCT^ method was used to calculate relative changes in gene expression.

### Quantification of histamine by ELISA

Wild‐type *L. reuteri* 6475 and 6475::*folC2* were grown as described above in LDMIII or LDMIII + 4 mg/mL l‐histidine. Cultures were harvested at 24 h, centrifuged (1500*g*), and filter‐sterilized with 0.22‐*μ*m PVDF filters. Histamine concentrations were determined as previously described (Thomas et al. 2012) using the Histamine ELISA kit (Neogen, Lexington, KY). Absorbance was measured with a Spectramax 340PC (Molecular Devices, Sunnyvale, CA), and data were analyzed using GraphPad Prism 5 software. Data were corrected with values obtained from the background control.

### Preparation of bacterial supernatants and administration to mice

Bacterial supernatants were prepared as described for the TNF inhibition bioassay above, filter‐sterilized, and concentrated 20× with speed vacuum drying. Administration to mice was as described previously (Hemarajata et al. 2013). In brief, each mouse received two intraperitoneal injections of bacterial supernatant or medium control, with the first dose at 18 h before TNBS rectal enema (described below) and the second dose at 2 min before TNBS enema. All mouse experiments were performed in a Specific Pathogen‐Free (SPF) animal facility, according to an Institutional Animal Care and Use Committee (IACUC)‐approved mouse protocol at Baylor College of Medicine, Houston, TX.

### Induction of acute colitis using TNBS rectal enema

Female Balb/c mice (45‐day old) were received from Harlan Laboratories (Houston, TX) and maintained under specific pathogen‐free conditions. Animals were provided standard chow and water and allowed to feed *ad libitum* under a 12 h daylight cycle. Mice acclimated post‐shipment for 10 d. Induction of TNBS colitis, determination of colitis severity, and protection conferred by probiotic compounds was performed according to established protocols with minor modifications as described previously (Foligne et al. 2006; Hemarajata et al. 2013). In brief, mice were anesthetized by constant isoflurane inhalation. A 5% v/v TNBS (Sigma‐Aldrich, St. Louis, MO) solution in water was diluted with equal volume of absolute ethanol and administered intrarectally via catheter at a dose of 100 mg/kg body weight, 4 cm distal to the anus. Mice were kept head down in a vertical position for 2 min after enema to ensure complete retention of enema in the colon. Procedure control mice received 50% ethanol in PBS as an enema and two IP injections of the medium control. Colitis‐positive mice received a TNBS enema and two IP injections of the medium control, while treated mice received a TNBS enema and two IP injections of the prepared bacterial supernatant. Mice were weighed immediately prior to TNBS enema and again 48 h after TNBS enema. Percent weight loss was calculated based on differences between these measurements.

### Macroscopic assessment of TNBS‐induced colitis

Colons were collected 48 h after induction of TNBS colitis and opened longitudinally. Colonic inflammation and damage were determined according to the Wallace criteria (Morris et al. 1989). In brief, the grading scale was: Score 0: normal/healthy appearance; Score 1: focal hyperemia, slight thickening, and no ulcers; Score 2: hyperemia, prominent thickening, and no ulcers; Score 3: ulceration with inflammation at one site; Score 4: ulceration with inflammation at two or more sites; Score 5: major sites of damage extending >1 cm; Score 6–10: when area of damage extends >2 cm, the score is increased by each additional cm of tissue involvement. Each colon was scored blindly by one individual.

### Plasma measurements of mouse serum amyloid protein A (SAA)

Blood samples were collected from mice via cardiac puncture, stored with anticoagulant, and centrifuged (10 min, 17000*g*) to isolate plasma. SAA levels in plasma were measured using ELISA kits from ALPCO (Salem, NH) according to the manufacturer's instructions.

## Results

### Identification of 5,10‐CH = THF polyglutamate compounds produced by TNF‐inhibitory *L. reuteri* 6475

**Figure 1:**
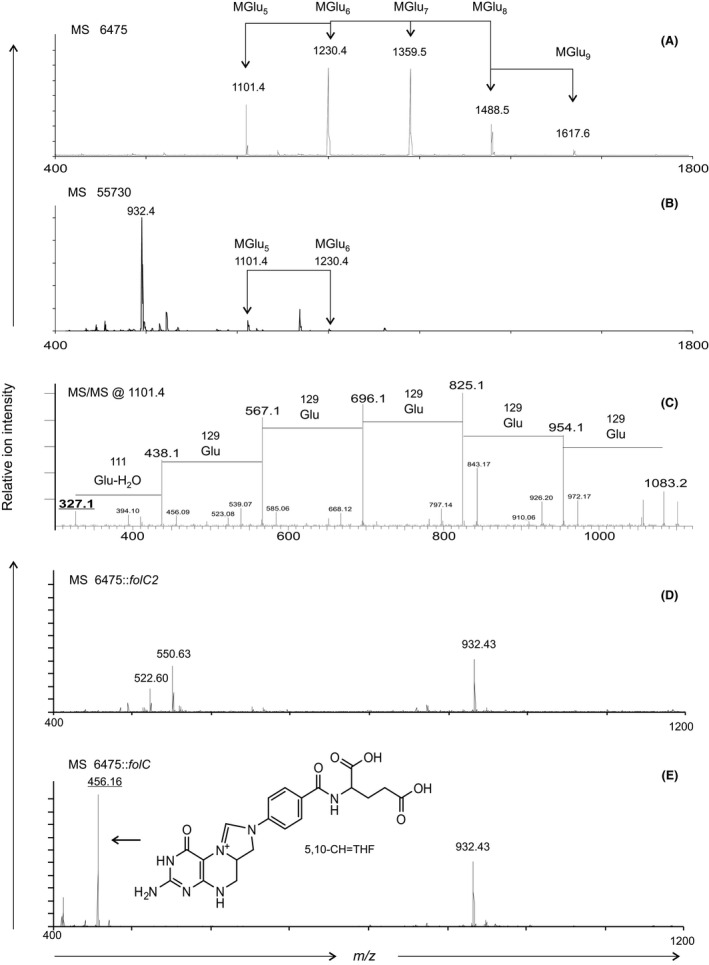
MALDI‐MS detection of 5,10‐CH = THF polyglutamates in TFA‐treated *L. reuteri* cell pellets. Peaks indicating the presence of 5,10‐CH = THF polyglutamate (arrows, MGlu_n_) are marked for strain (A) 6475 and (B) 55730. The subscripted number (*n*) after MGlu indicates the number of glutamate residues present in the polyglutamate tail, not counting the intrinsic glutamate of folic acid. (C) MALDI ion trap MS/MS fragmentation analysis of MGlu_5_ at *m/z* 1101.4 confirms the presence of a folate compound, consistent with the lightest MS/MS fragment ion at *m/z* values 327.1. (D) Inactivation of *folC2* in 6475 inhibited the production of 5,10‐CH = THF compounds as monitored by MALDI MS. (E) The 6475::*folC* mutant produced 5,10‐CH = THF (structure depicted), but no 5,10‐CH = THF polyglutamates of any chain length. The unidentified ion at *m/z* 932.4 was consistently observed after TFA treatment of all *L. reuteri* cell pellets. All spectra were scaled to comparable maximum intensity levels. TFA, trifluoroacetic acid; 5,10‐CH = THF, 5,10‐methenyltetrahydrofolic acid.

*L. reuteri* cell pellets from stationary phase cultures were treated with 0.1% trifluoroacetic acid (TFA)‐acidified water to collect a concentrated solution of extracellular compounds loosely associated with the bacterial cell surface. TFA‐treated cell pellets from TNF‐inhibitory *L. reuteri* 6475 and the non‐TNF‐inhibitory strain 55730 were analyzed by matrix‐assisted laser desorption/ionization time‐of‐flight (MALDI‐TOF) mass spectrometry. Differences in the composition of TFA‐treated cell pellets were observed. Multiple peaks (labeled MGlu_n_) differing by *m/z* 129, the expected mass of glutamate (Glu), were identified in strain 6475 (Fig. 1A). Two of these peaks were also identified in strain 55730, but the peak intensity was much less compared to strain 6475 (Fig. 1B). These peaks were also identified in bacterial cell‐free culture supernatants from strains 6475 and 55730 (data not shown). The observed masses did not match the predicted masses for simple polyglutamate homopolymers, indicating the presence of a covalently linked compound (M). MS/MS fragmentation analysis in both *L. reuteri* strains 6475 and 55730 at *m/z* 1101.4 (MGlu_5_) (Fig. 1C) indicated that the core compound M was 5,10‐methenyltetrahydrofolic acid (5,10‐CH = THF) with a covalently linked, polyglutamate homopolymer tail (5,10‐CH = THF polyglutamate). Neither 5,10‐CH = THF nor 5,10‐CH = THF polyglutamate were produced by the *folC2* mutant (Fig. 1D), and only 5,10‐CH = THF is produced by the *folC* mutant (Fig. 1E). The structure of 5,10‐CH = THF is depicted in Fig. 1E.

### The *folC2* gene in *L. reuteri* is required for human TNF suppression

**Figure 2:**
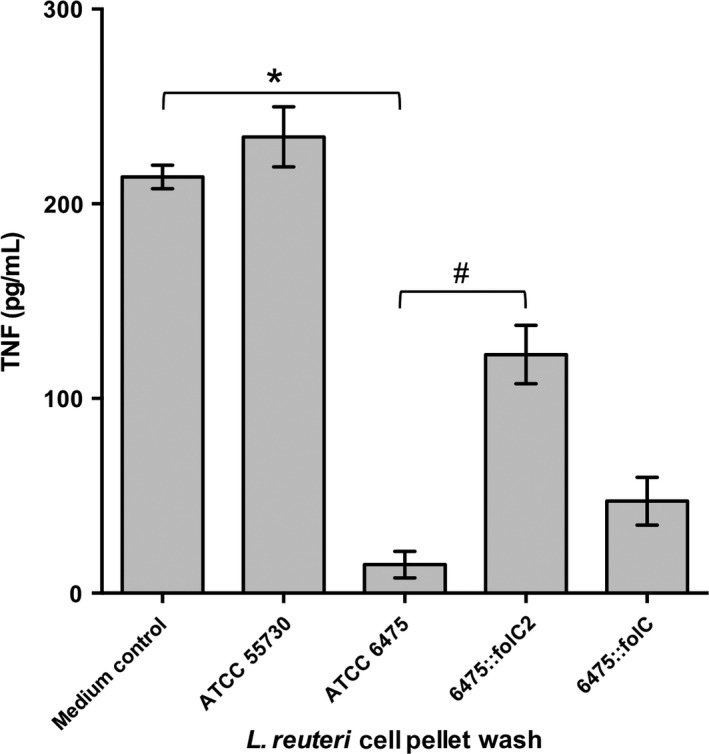
The *folC2* gene contributed to the TNF‐suppressive ability of *L. reuteri* 6475. TFA‐treated cell pellets (normalized to cell pellet weight of 1 g) from various *L. reuteri* strains were tested for the ability to inhibit TNF by TLR2‐activated THP‐1 cells. THP‐1 cells were treated with 100 ng/mL PCK (TLR2 agonist) in the presence of *L. reuteri* for 3.5 h, and TNF production was monitored by ELISA. Wild‐type 6475 significantly inhibited TNF compared to medium control. The 6475::*folC2* mutant yielded reduced capability to inhibit TNF production compared to wild‐type 6475. There was no significant difference between 6475 and 6475::*folC* strains. Data were analyzed with one‐way analysis of variance with Bonferroni's multiple comparison test correction, mean ± SD, *n* = 3, *P < 0.05 compared to medium control ^#^ *P* < 0.05 compared to 6475.TLR2, Toll‐like receptor 2; TNF, tumor necrosis factor.

Targeted mutagenesis was used to construct mutants defective in bifunctional dihydrofolate synthase/folylpolyglutamate synthase type 2 (*folC2*) and bifunctional dihydrofolate synthase/folylpolyglutamate synthase (*folC*), 6475::*folC2* and 6475::*folC*, respectively. The ability of the mutants to modulate TNF levels was compared to wild‐type strain 6475. Wild‐type *L. reuteri* 6475 TFA‐treated cell pellet washes containing 5,10‐CH = THF and 5,10‐CH = THF polyglutamate significantly inhibited TNF production by activated human monocytoid cells stimulated with a Toll‐like receptor 2 (TLR2) agonist (*P* < 0.05) (Fig. 2). Inactivation of *folC* had no significant effect on TNF inhibition by strain 6475 (Fig. 2); however, inactivation of *folC2* in strain 6475 resulted in abrogation of the TNF‐inhibitory phenotype (Fig. 2), suggesting that *folC2* is a part of the immunomodulatory gene network. Similar results were obtained with the bacterial cell‐free culture supernatants of the same *L. reuteri* strains (Fig. S1A), indicating that the immunomodulins were actively secreted and associated with the bacterial cell surface. In TLR2‐activated human monocytoid cells, TNF gene expression was decreased by *L. reuteri* 6475 compared to the medium control (Fig. S1B), confirming prior results that *L. reuteri* suppressed human TNF at the transcriptional level (Lin et al. 2008). The 6475::*folC2* mutant did not suppress TNF gene expression compared to the medium control (Fig. S1B), indicating that the *folC2* gene was important for human TNF suppression by *L. reuteri* 6475 in vitro.

### FolC2 is necessary for 5,10‐CH = THF and 5,10‐CH = THF polyglutamate production and immunomodulation

Production of 5,10‐CH = THF and 5,10‐CH = THF polyglutamate in *L. reuteri* 6475::*folC2* and 6475::*folC* was determined by MALDI‐TOF mass spectrometry. Analysis revealed the absence of both 5,10‐CH = THF and 5,10‐CH = THF polyglutamate in the 6475::*folC2* mutant, which lacks the TNF‐inhibitory phenotype (Fig. 1D). Analysis of the 6475::*folC* mutant, which retains the TNF‐inhibitory phenotype demonstrated the presence of 5,10‐CH = THF (*m/z* 456.2), but not 5,10‐CH = THF polyglutamate (Fig. 1E). Strain 6475::*folC* inhibited TNF production (Fig. 2 and S1A), indicating that polyglutamylation of 5,10‐CH = THF was not necessary for TNF inhibition by *L. reuteri*.

**Figure 3:**
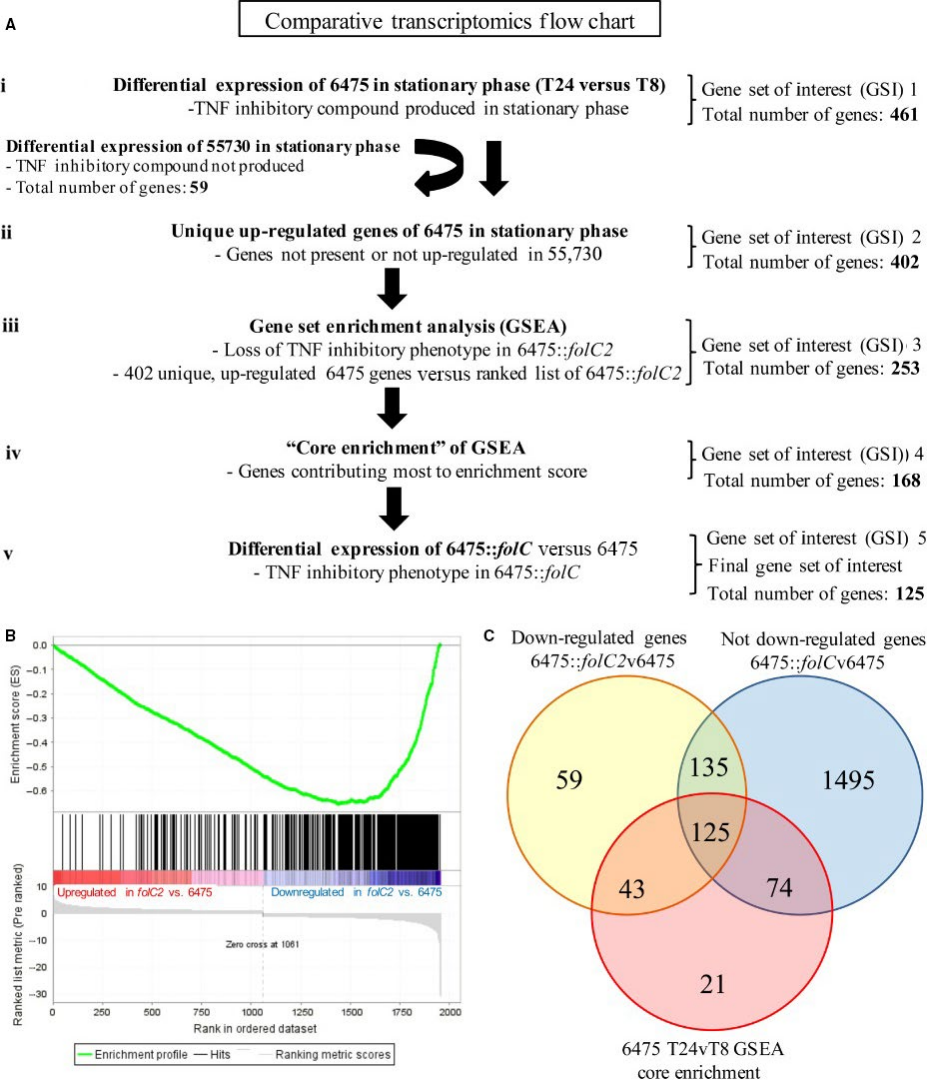
Comparative transcriptomics analysis revealed gene set encoding potential immunomodulins. (A) Flowchart of the comparative transcriptomic analysis employed in this study. *N* = 3 for each wild‐type and mutant *L. reuteri* strain that was included in the comparative analysis. (B) GSEA showed significant enrichment of strain 6475 genes up‐regulated in stationary phase in the down‐regulated genes of 6475::*folC2*. (C) The yellow circle indicates genes significantly down‐regulated in 6475::*folC2* that were up‐regulated in wild‐type 6475 (stationary phase). The blue circle indicates genes not down‐regulated in 6475::*folC* that were up‐regulated in wild‐type 6475 (stationary phase). The red circle indicates genes that were included in the GSEA “core enrichment.” The overlap between these three gene sets revealed a final gene set of interest including 125 genes potentially involved in immunomodulin production by *L. reuteri* 6475. GSEA, A Gene Set Enrichment Analysis

Additional *L. reuteri* genes potentially involved in immunomodulation were identified by comparative transcriptomics between wild‐type and mutant *L. reuteri* strains 6475 and 55730, representing the two known human clades II and VI, respectively (Spinler et al. 2014) (Fig 3A). *L. reuteri* immunomodulins (TNF‐inhibitory factors) were only detected in strain 6475 cultures grown to stationary phase (Lin et al. 2008). The gene expression profile of strain 6475 in stationary phase (24 h) was compared to the same strain in early log phase (8 h), and 461 significantly up‐regulated genes (*P* < 0.05) were identified as genes potentially important for immunomodulin production (Saulnier et al. 2011). These 461 up‐regulated genes comprised the initial gene set of interest. *L. reuteri* 55730 does not inhibit TNF, allowing the gene set of interest to be restricted to up‐regulated genes that were unique to strain 6475 or not significantly up‐regulated in strain 55730 in stationary phase (402 total genes). A Gene Set Enrichment Analysis (GSEA) demonstrated that these 402 genes were significantly enriched in 6475::*folC2* down‐regulated genes (*P* < 0.001, Fig. 3B) (Subramanian et al. 2005). Analysis stringency was increased by including only “core enrichment” genes, genes that contributed most to the GSEA enrichment score (168 genes). The list of immunoregulatory genes was further restricted by selecting only those genes not significantly down‐regulated in 6475::*folC* (24 h) versus wild‐type 6475 (24 h), since the *folC* mutant retained the ability to inhibit TNF production. The comparative transcriptomics analysis revealed a total of 125 potential immunoregulatory genes out of 1974 protein‐coding genes (Fig. 3A and 3C). These 125 genes (approximately 6% of the genome) were (1) significantly up‐regulated in strain 6475 grown to stationary phase, (2) not present or not significantly up‐regulated in strain 55730 grown to stationary phase, (3) significantly down‐regulated in 6475::*folC2*, (4) part of the GSEA “core enrichment,” and (5) not significantly down‐regulated in 6475::*folC* (Table S1). The 10 genes with the greatest fold change in 6475 stationary phase and 6475::*folC2* versus wild‐type 6475 (stringent selection) are listed in Table 1. The gene encoding the histidine/histamine antiporter (*hdcP*) was included in this exclusive list of top 10 genes and the gene encoding histidine decarboxylase pyruvoyl type A (*hdcA*) was included in the final list of 125 genes following GSEA. The *hdc* gene cluster was identified by global gene expression studies including folate pathway mutants, and mechanistic studies probing the ability of *Lactobacillus*‐generated histamine to suppress the proinflammatory human cytokine TNF (Thomas et al. 2012).

**Table 1:** Bacterial genes of interest

Gene ID	Description	Functional Group
NT01LR1336	Esterase	Central intermediary metabolism
NT01LR1981	Lr1016	Unclassified
NT01LR0282	Conserved hypothetical protein	Hypothetical protein
NT01LR1905	Conserved membrane protein	Cell envelope
NT01LR0849	Hypothetical protein	Hypothetical protein
NT01LR0279	Conserved hypothetical protein	Hypothetical protein
NT01LR1786	Respiratory nitrate reductase, *γ* subunit	Energy metabolism
NT01LR0128	Amidohydrolase family, putative	Central intermediary metabolism
NT01LR1242	Histidine/histamine antiporter	Transport and binding proteins
NT01LR1034	Hypothetical protein	Hypothetical protein

^1^The differential expression via distance synthesis (DEDS) statistic represents the selected differential expression measures from the previous analyses as a multivariate point cloud on as many dimensions as there are input statistics. The scalar distance to the most extreme combination of these input statistics, following permutation, was the final metric by which genes are ranked and displayed here.

### Expression of the *hdc* gene cluster and histamine production were diminished in *L. reuteri* 6475::*folC2*

**Figure 4:**
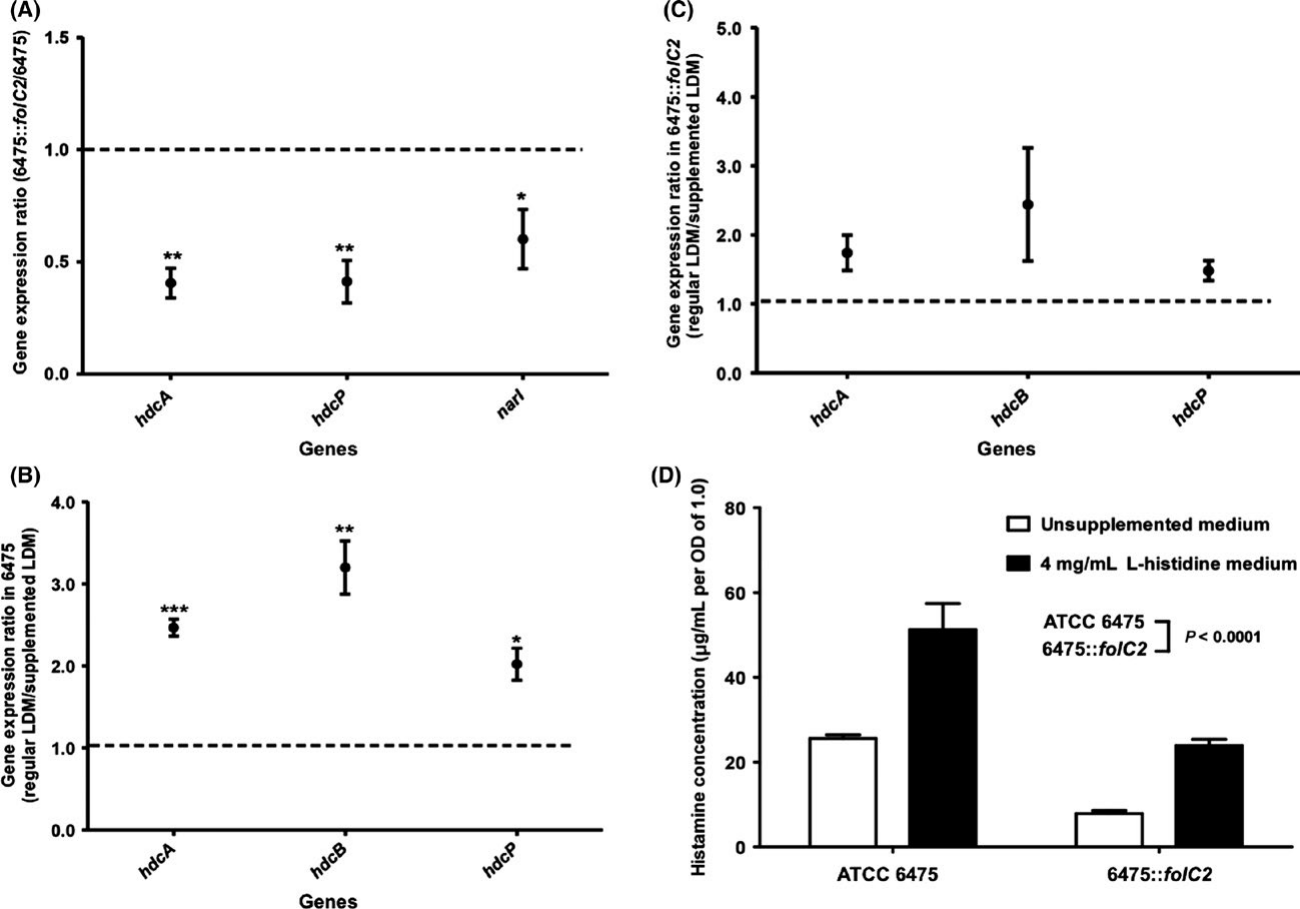
Inactivation of the *folC2* gene resulted in repression of the *hdc* gene cluster. (A) Quantitative real‐time PCR yielded evidence of decreased expression of *hdcA* and *hdcP* genes in the *L. reuteri* 6475::*folC2* mutant compared to wild‐type *L. reuteri* 6475. The gene *narI*, which was suggested by the comparative transcriptomics analysis to be down‐regulated in the *folC2* mutant, was also repressed in 6475::*folC2*. Expression ratios of each gene (*folC2* mutant vs. wild‐type) were calculated, and results represent the mean ± SD, *n* = 3, **P < 0.01, *P < 0.05 compared to the theoretical mean of 1.0. (B) Quantitative real‐time PCR demonstrated increased expression of all three *hdc* genes, *hdcA*,* hdcB*, and *hdcP*, when *L. reuteri* 6475 was grown in medium supplemented with l‐histidine compared to unsupplemented medium. Expression ratios of each gene (l‐histidine‐supplemented vs. unsupplemented) were calculated. Results represent the mean ± SD, *n* = 3, ***P < 0.005, **P < 0.01, *P < 0.05 compared to the theoretical mean of 1.0. (C) Quantitative real‐time PCR demonstrated no significant changes in expression of all three *hdc* genes when *L. reuteri* 6475::*folC2* was grown in medium supplemented with l‐histidine compared to unsupplemented medium. (D). Inactivation of the *folC2* gene resulted in decreased histamine production. Quantification of secreted *L. reuteri*‐derived histamine by a histamine‐specific ELISA demonstrated decreased histamine production in the *folC2* mutant compared to wild‐type *L. reuteri* 6475 even when grown in l‐histidine‐supplemented medium. Data were analyzed by two‐way ANOVA. Results represent the mean ± SD, *n* = 3, *P* < 0.0001 compared to wild‐type 6475.

Comparative transcriptomics analysis identified 125 genes that may be responsible for immunomodulation by *L. reuteri* 6475 (Table 1 and Table S1). Two identified genes, *hdcA* and *hdcP*, are *L. reuteri* genes known to be involved in conversion of histidine to histamine, a compound that suppresses TNF production by myeloid cells (Thomas et al. 2012). Quantitative RT‐PCR validated the comparative transcriptomics studies by confirming changes in gene expression in 6475::*folC2* for three of the 10 genes listed in Table 1: *hdcA*,* hdcP*, and respiratory nitrate reductase gamma subunit (*narI*). All three genes had diminished gene expression in the 6475::*folC2* mutant compared to wild‐type 6475 (Fig. 4A). Expression of the complete *hdc* gene cluster (*hdcA, hdcB,* and *hdcP*) was examined in wild‐type *L. reuteri* and 6475::*folC2*. Expression of the entire *hdc* gene cluster was increased in wild‐type *L. reuteri* 6475 grown in l‐histidine‐supplemented medium compared to unsupplemented medium (Fig. 4B), while expression was not significantly changed in 6475::*folC2* in the presence of additional histidine (Fig. 4C). Histamine production by *L. reuteri* 6475 and 6475::*folC2* strains was measured with a histamine‐specific ELISA. Production of histamine was significantly increased in *L. reuteri* 6475 when grown in medium supplemented with l‐histidine (Fig. 4D). The *folC2* mutant produced significantly less histamine compared to wild‐type *L. reuteri* even in the presence of media supplementation with l‐histidine (Fig. 4D).

### The *folC2* gene contributes to suppression of intestinal inflammation by *L. reuteri* 6475 *in vivo*

**Figure 5:**
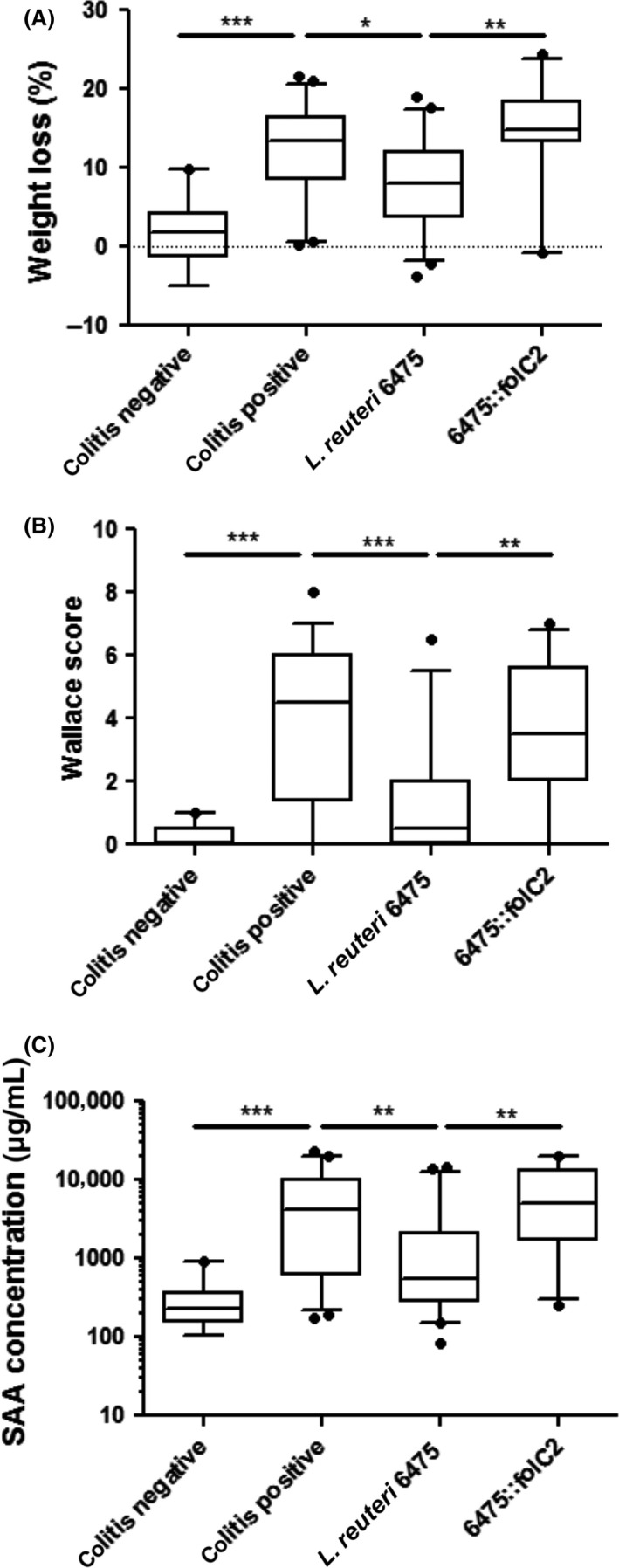
The *folC2* gene was essential for colitis attenuation and anti‐inflammatory activity of *L. reuteri* 6475 in vivo. (A) Supernatant from *L. reuteri* 6475 significantly decreased weight loss in mice challenged with intrarectal TNBS, whereas 6475::*folC2* did not have such effects. Differences in weight loss are shown as percent weight loss 48 h after induction of TNBS colitis. (B) Supernatant from *L. reuteri* 6475 significantly decreased colonic macroscopic injury in mice challenged with TNBS, whereas 6475::*folC2* did not have such effects. Differences in colonic macroscopic injury are shown as Wallace score. Data presented as mean ± SEM. (C) Supernatant from *L. reuteri* 6475 significantly decreased plasma SAA concentrations in mice challenged with TNBS, whereas 6475::*folC2* did not have such effects. Plasma levels of SAA, an indicator of inflammation, were measured by ELISA. *N* = 19, 50, 47, and 26 for colitis‐negative, colitis‐positive, ATCC 6475, and 6475::*folC2*, respectively. Statistical analyses were performed using GraphPad Prism (GraphPad Inc., La Jolla,CA). Data were presented using box and whisker plots showing the median and 5th and 95th percentiles. Statistical significance was assessed by nonparametric Kruskal–Wallis test. Differences between experimental groups are reported as mean fold difference ± SEM, ***P < 0.001, **P < 0.01, *P < 0.05.SAA, Serum amyloid A; TNBS, trinitrobenzene sulfonic acid.

To investigate whether the *folC2* gene contributes to anti‐inflammatory effects *in vivo*,* L. reuteri* strain 6475 and 6475::*folC2* bacterial cell‐free supernatants were tested in a TNBS‐induced mouse model of acute colitis. An 8‐week‐old female inbred Balb/c mice received two intraperitoneal (IP) injections of concentrated bacterial supernatant (18 h apart) followed by induction of colitis by TNBS instillation. Mice that received IP injections of the medium control and were challenged with TNBS (colitis‐positive mice) or phosphate‐buffered saline (PBS) (colitis‐negative mice) were studied as controls. Weight loss, which reflects the overall health status of mice, and a Wallace scoring system, which assesses the relative extent of macroscopic colon injury and inflammation, were measured to evaluate colitis severity. Serum amyloid A (SAA), an acute phase protein that serves as a plasma biomarker of intestinal mucosal inflammation in mice and correlates with severity of colitis, (de Villiers et al. 2000; Uhlar and Whitehead 1999) was quantified in plasma. Colitis‐negative mice showed no evidence of spontaneous disease (Fig. 5A–B). Colitis‐positive mice developed moderate colitis characterized by weight loss and macroscopic intestinal inflammation (Fig. 5A–B). As expected, these mice had significantly elevated concentrations of SAA (*P* < 0.001) compared to colitis‐negative mice (Fig. 5C). IP injection with *L. reuteri* 6475 supernatant reduced macroscopic inflammation (Fig. 5B). Treatment with *L. reuteri* 6475 supernatant also resulted in diminished weight loss 48 h post‐treatment compared to colitis‐positive mice (Fig. 5A), and reduced quantities of SAA in peripheral blood serum (Fig. 5C). Treatment with bacterial supernatants from the *L. reuteri* 6475::*folC2* strain did not attenuate colitis as indicated by no significant change in body weight or macroscopic colonic inflammation (Fig. 5A–B). Additionally, the 6475::*folC2* supernatant did not reduce SAA compared to colitis‐positive mice (Fig. 5C).

## Discussion

**Figure 6:**
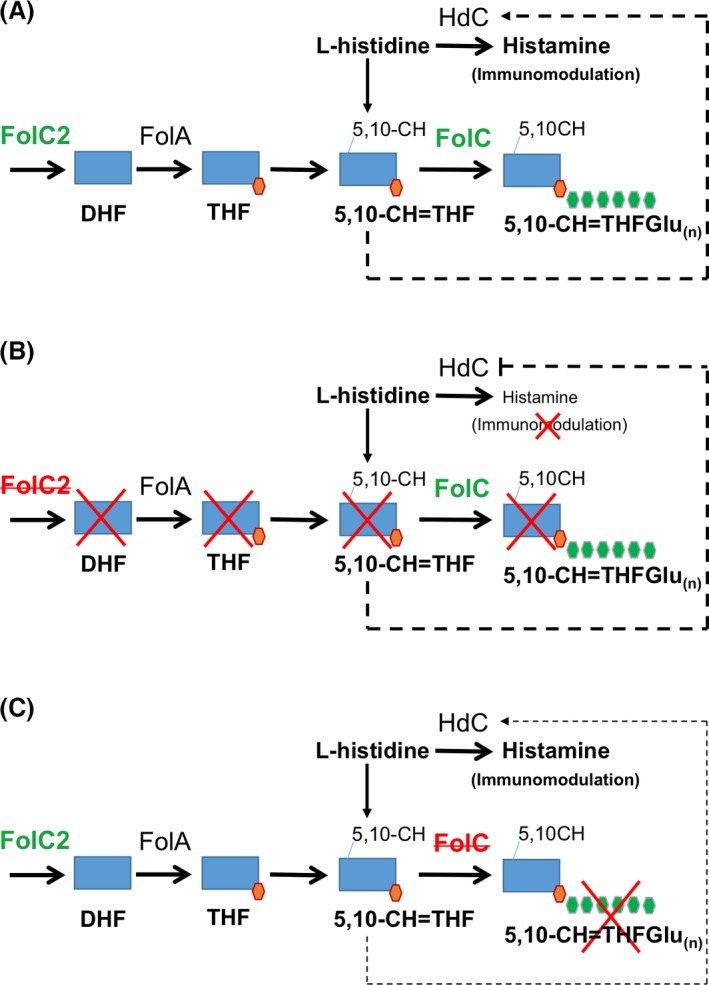
Folate synthesis appears to be linked to histamine production in *L. reuteri* 6475. (A) Folate synthesis in wild‐type *L. reuteri* 6475 with FolC2 necessary for production of dihydrofolate and FolC responsible for addition of a polyglutamate tail to tetrahydrofolate. Folate synthesis contributes to histamine production and the anti‐inflammatory effect of *L. reuteri*. (B) Inhibition of folate synthesis in *L. reuteri* 6475::*folC2* leads to reduced histamine production and loss of anti‐inflammatory activity. (C) Inhibition of folate polyglutamylation in *L. reuteri* 6475::*folC* does not impact histamine production and *L. reuteri* anti‐inflammatory activity is preserved. Hdc, histidine decarboxylase.

Probiotic *Lactobacillus* species secrete a variety of organic compounds that may regulate host immune responses (Lin et al. 2008). Our results show that specific strains of *L. reuteri* are capable of producing abundant 5,10‐CH = THF and 5,10‐CH = THF polyglutamate compounds. This report clarifies the contributions of the *folC* and *folC2* genes to folate metabolism in Firmicutes. While detection of 5,10‐CH = THF and polyglutamylated 5,10‐CH = THF have been reported in other lactic acid bacteria cultures (Sybesma et al. 2003), this study demonstrates that production of these compounds in human‐derived *L. reuteri* strains correlates with the ability to inhibit TNF production *in vitro*. Mutagenesis of the *L. reuteri folC2* gene and subsequent lack of 5,10‐CH = THF is associated with loss of TNF‐inhibitory activity *in vitro*. *L. reuteri* 6475 attenuates murine TNBS‐induced colitis and the *folC2* gene is necessary for *L. reuteri's* protective and anti‐inflammatory activities *in vivo*. Additional studies demonstrate decreased *L. reuteri hdc* gene expression and histamine production in 6475::*folC2*, linking the pathways responsible for 5,10‐CH = THF production to histamine production and immunomodulation (Fig. 6A–B).

This manuscript provides insights into genes and pathways involved in folate metabolism by human‐associated microbes. Varied chain length 5,10‐CH = THF polyglutamates (up to nine glutamate residues) were identified in cell wall‐associated compounds and secreted factors of TNF‐inhibitory *L. reuteri* strain 6475. Production of 5,10‐CH = THF and 5,10‐CH = THF polyglutamate of any chain length by strain 6475 required *folC2*. In contrast, 6475::*folC* maintained production of 5,10‐CH = THF but without the polyglutamate tail. Based on these results, *folC2* appears to be necessary for production of 5,10‐CH = THF, and *folC* appears to encode the enzyme involved in polyglutamylation of 5,10‐CH = THF in *L. reuteri*. To date, activities of FolC2 have not been well characterized in lactobacilli. FolC2 and FolC are not orthologs and are included in different Clusters of Orthologous Groups (COGs), COG1478 and COG0285, respectively (de Crecy‐Lagard 2014). However, both FolC and FolC2 are considered to be bifunctional enzymes with dihydrofolate synthase and folylpolyglutamate synthase activity (Bognar and Shane 1986; Toy and Bognar 1990; Kimlova et al. 1991; Murata et al. 2000). Dihydrofolate synthase is needed for the synthesis of dihydrofolate by coupling glutamate with dihydropteroate. Conversion of dihydrofolate to tetrahydrofolate (THF) is performed as an intermediate reaction by dihydrofolate reductase, which is encoded by the *folA* gene (Fig. 6A). Presence of *folA* is ubiquitous in *Lactobacillus* strains (Magnusdottir et al. 2015), and consequently should not play a major role in differential folate production in lactobacilli. Folylpolyglutamate synthase adds a glutamyl tail to THF (Fig. 6A). Many lactobacilli possess *folC* in their genome, however, prevalence of *folC2* is restricted to fewer species (PATRIC database, https://www.patricbrc.org/). Whether or not these genes are fully functional in all species and strains is currently unknown. *FolC2* is found in human‐derived *Lactobacillus* strains (*L. reuteri, L. iners)*, as well as in lactobacilli commonly found in fermented foods (*L. plantarum*,* L. delbrueckii*,* L. sakei, L. pentosus, L. buchneri,* and *L. hilgardii*). These genes have been poorly characterized at the functional level and more research is needed in this area.

The 5,10‐CH = THF compound that we detected in *L. reuteri* 6475 can be synthesized in two successive reactions from THF (Fig. 6A). THF is converted to 10‐formyl tetrahydrofolate (10‐CHO = THF) via formate tetrahydroligase. 10‐CHO = THF is then transformed into 5,10‐CH = THF by the bifunctional enzyme methylenetetrahydrofolate dehydrogenase/methenyltetrahydrofolate cyclohydrolase. The genes encoding these two enzymes are present in the *L. reuteri* strains included in this report. The conversion of 10‐CHO = THF into 5,10‐CH = THF also occurs spontaneously at a very low pH (Eto and Krumdieck 1980; Arnold and Reilly 2000), however, our data suggest that 5,10‐CH = THF was synthesized by *L. reuteri*.

The generation of tetrahydrofolate compounds by *L. reuteri* strains contributes to immunoregulation (TNF suppression) through interconnected metabolic pathways involved in folate and histidine metabolism. Even if lactobacilli can produce abundant, long‐chain 5,10‐CH = THF, the TNF‐inhibitory phenotype relies primarily on the ability of specific strains to convert l‐histidine to histamine and several histamine metabolites (Spinler et al. 2014). Folate compounds mediate the interconversion of serine and glycine, and play a role in histidine biosynthesis and catabolism (Broquist 1957). The *folC2* gene may exert its immunomodulatory effects by regulating the production of histamine, a known *L. reuteri* immunomodulin (Figs 6A and B). The *folC* gene plays a role in adding the polyglutamate tail to 5,10‐CH = THF, but unlike *folC2*, this gene does not affect the production of histamine nor the ability of *L. reuteri* to suppress production of proinflammatory cytokines (Fig. 6C). Histamine production as well as the expression of genes involved in histamine production were decreased in the 6475::*folC2* mutant, and parallel changes in 5,10‐CH = THF and histamine production suggest that folate metabolism and specifically the *folC2* gene may be important for the conversion of l‐histidine to histamine. The synchronous changes in gene expression affecting histamine and folate metabolism link these two pathways, contributing to the immunomodulatory phenotype of *L. reuteri*. Further studies will continue to elucidate biologic connections between histamine, a known TNF‐inhibitory factor, l‐histidine metabolism, and other compounds produced by *L. reuteri* 6475 including 5,10‐CH = THF.

Tetrahydrofolic acid and its derivatives (5,10‐CH = THF, 10‐CHO = THF) are essential cofactors that facilitate the transfer of single‐carbon units from donor molecules into important biosynthetic pathways leading to methionine, purine, and pyrimidine biosynthesis (Ragsdale 2008; Fowler 2001). Histidine is a known end product of purine biosynthesis (Allen et al. 2002). Additionally, the oxidation of amino acids, including histidine, has been linked to the role of folate in generating reducing agents, NADH and NADPH, in eukaryotes (Brosnan et al. 2015). Our gene expression data suggest that this function of folate reviewed by Brosnan et al. may also occur in prokaryotes. In the *folC2* mutant, there is down‐regulation of several genes encoding enzymes that require NADPH as a substrate (i.e., thioredoxin reductase, nitrate reductase, methionine‐S‐reductase). These enzymes are involved in oxidative stress response, making it possible that the redox state of *L. reuteri* plays a role in its immunosuppressive function. Preliminary studies demonstrate that growth of *L. reuteri* in anaerobic versus aerobic conditions (different redox states) affects folate production and immunomodulation. The studies presented here were performed under standard anaerobic conditions. When *L. reuteri* is grown under aerobic conditions, the composition of folate compounds is different and the ability of *L. reuteri* to inhibit TNF production is reduced (data not shown). Further studies are needed to understand the metabolic pathways of *L. reuteri* and how they are regulated to produce histamine and 5,10‐CH = THF, resulting in the immunosuppression phenotype of *L. reuteri*.

Secreted factors produced by wild‐type *L. reuteri* 6475 can significantly ameliorate the intestinal pathology and inflammation in a TNBS‐induced mouse model of colitis. Previous studies demonstrated that delivery of pharmacological agents via the IP route is an efficient and successful method, especially when the target is within the peritoneal cavity (Chaudhary et al. 2010). We administered *L. reuteri*‐derived secreted factors in a concentrated form inside the peritoneal cavity and observed significantly diminished ulceration, local inflammation, and mucosal biomarkers of inflammation. Our study demonstrates that secreted factors produced by *L. reuteri* are important for preventing colitis, enabling alternative strategies that could supplant or supplement delivery of intact viable bacteria or bacterial colonization in the gut. These findings have important implications for future human clinical trials. Studies of various *L. reuteri* mutants in this model have provided insights into candidate genes that may be essential for suppression of intestinal inflammation by the model commensal *L. reuteri* strain 6475. In this study, we demonstrated the role of the *Lactobacillus* gene, *folC2*, as a potential key regulatory gene in the microbiome involved in protection from or amelioration of colitis. Our data support the conclusion that *L. reuteri* strain 6475 protects mammals from severe intestinal inflammation by production and secretion of potent immunosuppressive compounds locally in the gut lumen.

Deficiencies in bacterial‐derived micronutrients such as folate can lead to immune dysregulation highlighting the interdependence between diet, commensal bacteria, and the host mucosal immune system (Spencer and Belkaid 2012). Diminished TNF production by M1 macrophages has been observed when these cells were grown in the presence of folic acid (Samaniego, 2014). Immune cells of the GI tract such as regulatory T cells express high levels of the folate receptor 4 (FR4) (Yamaguchi et al. 2007). Interestingly, it has been demonstrated that folic acid can prevent TNBS‐induced colitis by maintaining regulatory T cells via suppression of apoptosis and subsequent prevention of colonic inflammation. Mice fed a folic acid‐free diet had significantly increased colonic inflammation, weight loss, and mortality rate after exposure to TNBS compared to mice fed a normal chow diet (Kinoshita et al. 2012). Protection against colitis could be restored if FR4‐expressing regulatory T cells were transferred into folic acid‐free diet mice prior to induction of colitis (Kinoshita et al. 2012). Folate is also able to activate mucosal‐associated invariant T cells (MAIT) in the GI tract by acting as antigens on infected cells (Chua and Hansen 2012; Kjer‐Nielsen et al. 2012). In our TNBS‐induced colitis studies, decreased production of 5,10‐CH = THF by *L. reuteri* 6475::*folC2* may contribute to the diminished protective effect by reduced TNF suppression as well as loss or dysfunction of regulatory T cells in the colon.

A comprehensive understanding of gene networks and gene regulation in beneficial gut microbes is critical to understanding the interaction of bacterial metabolites with the host. This knowledge may enhance the ability of the scientific community to select or engineer commensal gut bacterial strains that can suppress mucosal inflammation. Combining a genome‐scale metabolic model of *Bacteroides thetaiotaomicron* (iAH991) with a mouse metabolic model demonstrated the essential host–microbe symbiosis that occurs in the GI tract (Heinken et al. 2013). Modeling metabolic interactions between a gut microbe and its host has enabled identification of metabolites that are exchanged between the two organisms, influences on growth fitness for both organisms, and the ability of commensal bacteria to rescue lethal enzyme deficiencies in the host (Heinken et al. 2013). The recent identification of multiple biological pathways and genes involved in suppression of proinflammatory cytokine production in *L. reuteri* provides opportunities for combining such discoveries in future therapies and disease prevention strategies. For example, the potential dietary contribution of the amino acid l‐histidine to bacterial histamine generation in combination with methods to enhance production of tetrahydrofolic acid compounds may result in nutritional and immunomodulatory benefits for the mammalian host. As metabolic pathways and modules become linked together in human microbiome research (Hmp 2012), nutritional and medical interventions may promote healthy whole body metabolism and immune function in partnership with the gut microbiome. Future probiotic strategies will benefit from the identification of biochemical compounds and genes required for healthy intestinal physiology and probiotic‐mediated immunomodulation.

## Conflict of Interest

JV receives unrestricted research support from BioGaia AB.

## Supporting information


**Figure S1.** *FolC2* was necessary for suppression of TNF production at protein and mRNA levels. (A) *L. reuteri* cell‐free supernatants (normalized to an OD_600_ of 1.5) were tested for the ability to inhibit TNF production by TLR2‐activated THP‐1 cells. THP‐1 cells were treated with 100 ng/mL PCK (TLR2 agonist) in the presence of *L. reuteri* for 3.5 h and TNF production was monitored by ELISA. As seen with the cell pellets, wild‐type 6475 significantly inhibited TNF compared to medium control. The 6475::*folC2* mutant yielded significantly reduced ability to inhibit TNF production compared to wild‐type 6475. There was no significant difference between 6475 and 6475::*folC* in terms of effects on human TNF production. Data were analyzed with one‐way analysis of variance with Bonferroni's multiple comparison test correction, mean ± SD, *n* = 3, *P < 0.05 compared to medium control ^#^ *P* < 0.05 compared to 6475. (B) TNF gene expression was determined in THP‐1 cells treated with a TLR2 agonist plus medium control, 6475, or 6475::*folC2* cell‐free supernatants. Quantitative real‐time PCR demonstrated down‐regulation of human TNF gene expression by *L. reuteri* strain 6475. No significant effects on human TNF gene expression were seen when THP‐1 cells were treated with 6475::*folC2*. Gene expression data were normalized using five housekeeping genes, *b2 m, hprt1, rpl13A, gapdh,* and *actb*. Expression ratios of *tnf* (*L. reuteri* strain/medium control) were calculated, and results represent the mean ± SD, *n* = 3, *P < 0.05 compared to the theoretical mean of 1.0.

**Table S1.** Final Gene Set of Interest – 125 genes potentially involved in immunomodulation by wild‐type 6475.

**Table S2.** Bacterial strains, vectors, and primers used in this study.
