# Supplementary figures and images for: FolC2‐mediated folate metabolism contributes to suppression of inflammation by probiotic Lactobacillus reuteri
Source: Microbiologyopen. 2016 Jun 28;5(5):802–18. doi: 10.1002/mbo3.371 (PMC5061717; doi:10.1002/mbo3.371)

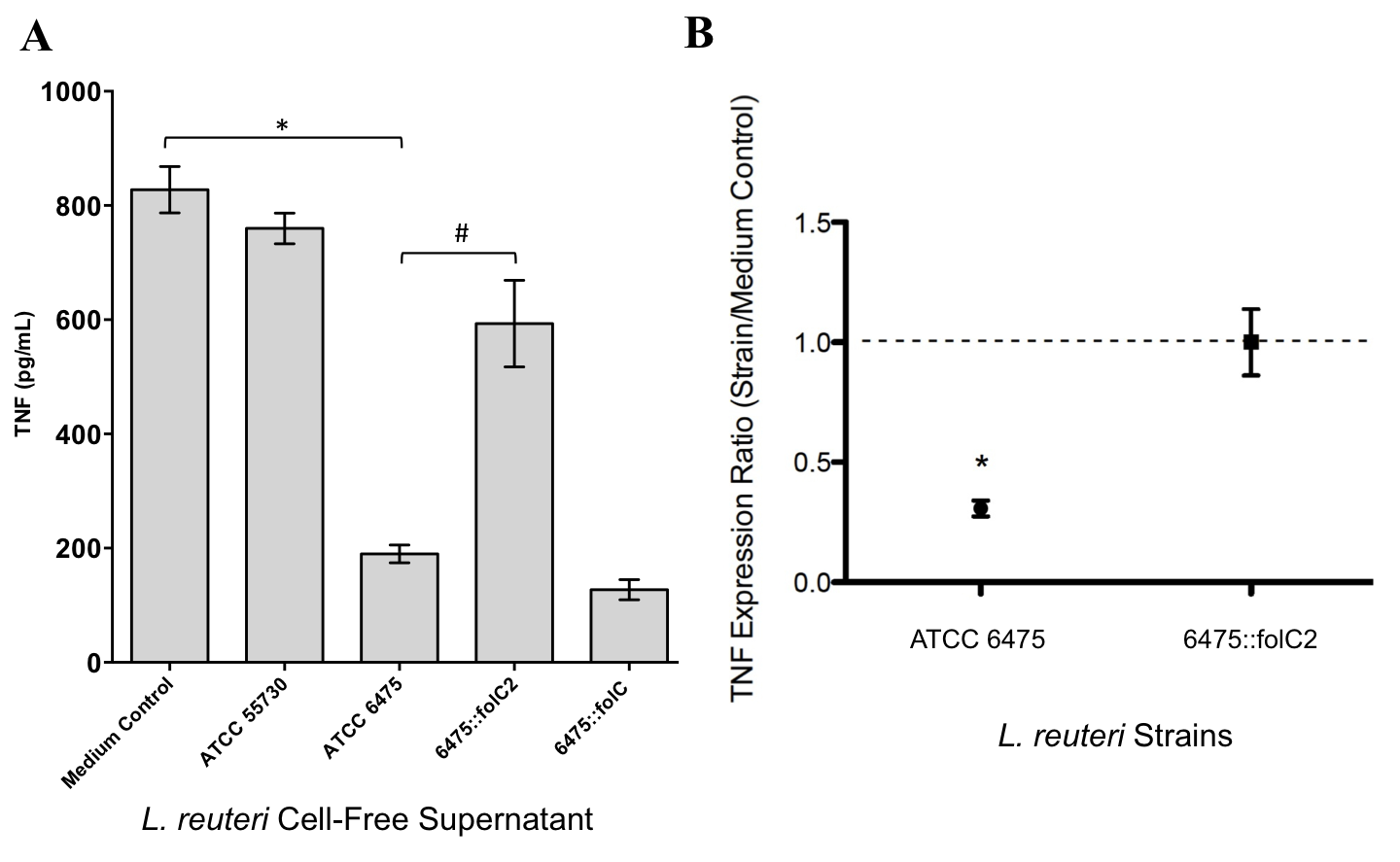

Supplement: Supplementary file 1 — Figure S1. FolC2 was necessary for suppression of TNF production at protein and mRNA levels. (A) L. reuteri cell‐free supernatants (normalized to an OD600 of 1.5) were tested for the ability to inhibit TNF production by TLR2‐activated THP‐1 cells. THP‐1 cells were treated with 100 ng/mL PCK (TLR2 agonist) in the presence of L. reuteri for 3.5 h and TNF production was monitored by ELISA. As seen with the cell pellets, wild‐type 6475 significantly inhibited TNF compared to medium control. The 6475::folC2 mutant yielded significantly reduced ability to inhibit TNF production compared to wild‐type 6475. There was no significant difference between 6475 and 6475::folC in terms of effects on human TNF production. Data were analyzed with one‐way analysis of variance with Bonferroni's multiple comparison test correction, mean ± SD, n = 3, *P < 0.05 compared to medium control # P < 0.05 compared to 6475. (B) TNF gene expression was determined in THP‐1 cells treated with a TLR2 agonist plus medium control, 6475, or 6475::folC2 cell‐free supernatants. Quantitative real‐time PCR demonstrated down‐regulation of human TNF gene expression by L. reuteri strain 6475. No significant effects on human TNF gene expression were seen when THP‐1 cells were treated with 6475::folC2. Gene expression data were normalized using five housekeeping genes, b2 m, hprt1, rpl13A, gapdh, and actb. Expression ratios of tnf (L. reuteri strain/medium control) were calculated, and results represent the mean ± SD, n = 3, *P < 0.05 compared to the theoretical mean of 1.0. [file MBO3-5-802-s001.tiff]
