# Supplementary material for: FolC2‐mediated folate metabolism contributes to suppression of inflammation by probiotic Lactobacillus reuteri
Source: Microbiologyopen. 2016 Jun 28;5(5):802–18. doi: 10.1002/mbo3.371 (PMC5061717; doi:10.1002/mbo3.371)
Supplement: Supplementary file 2 — Table S1. Final Gene Set of Interest – 125 genes potentially involved in immunomodulation by wild‐type 6475. [file MBO3-5-802-s002.docx]

**Supplemental Table S1. Final Gene Set of Interest – 125 genes potentially involved in immunomodulation by wild-type 6475**

| **Functional Group and Gene ID** | **Description** |
| --- | --- |
| **Central intermediary metabolism** |  |
| NT01LR1336 | Esterase |
| NT01LR0128 | Amidohydrolase family, putative |
| NT01LR0613 | Bacilysin biosynthesis oxidoreductase BacC |
| NT01LR1012 | D-lactate dehydrogenase |
| NT01LR1038 | Thioredoxin reductase |
| NT01LR0252 | Methyltransferase |
| NT01LR0268 | Low molecular weight protein tyrosine phosphatase YfkJ |
| **Unclassified** |  |
| NT01LR1981 | lr1016 |
| NT01LR0091 | lr1801 |
| NT01LR1241 | YitW |
| **Cell envelope** |  |
| NT01LR1905 | Conserved membrane protein |
| NT01LR1107 | Lipoprotein, putative |
| NT01LR2008 | Cell surface protein |
| NT01LR1842 | lpxtg-motif cell wall anchor domain protein |
| **Energy metabolism** |  |
| NT01LR1786 | Respiratory nitrate reductase, gamma subunit |
| NT01LR0202 | Dihydrolipoyllysine residue acetyltransferase component of pyruvate dehydrogenase |
| NT01LR0201 | Pyruvate dehydrogenase E1 component subunit beta |
| NT01LR1071 | Glucose-6-phosphate isomerase |
| NT01LR1789 | Nitrate reductase, alpha subunit |
| NT01LR1402 | Beta galactosidase small chain |
| NT01LR1282 | Multimeric flavodoxin WrbA family protein |
| NT01LR0203 | Dihydrolipoyl dehydrogenase |
| NT01LR0720 | NAD(P)H-dependent FMN reductase LOT6 |
| NT01LR1243 | Histidine decarboxylase, pyruvoyl type |
| NT01LR1966 | Triose-phosphate isomerase |
| NT01LR1401 | Beta-galactosidase large subunit |
| NT01LR1264 | Zinc-binding alcohol dehydrogenase family protein |
| NT01LR0667 | Phosphomannose isomerase type I |
| NT01LR1454 | UDP-glucose 4-epimerase |
| NT01LR1482 | Thioredoxin |
| NT01LR0639 | Cysteine-rich domain of 2-hydroxy-acid oxidase GlcF |
| **Transport and binding proteins** |  |
| NT01LR1242 | Histidine/histamine antiporter |
| NT01LR1032 | Amino acid transporter |
| NT01LR0735 | Arabinose efflux permease |
| NT01LR1210 | Amino acid permease |
| NT01LR1273 | Permease of the major facilitator superfamily |
| NT01LR0135 | Cation transport ATPase |
| **Cellular processes** |  |
| NT01LR1015 | Transcriptional regulator, TetR family |
| NT01LR1982 | Copper translocating P-type ATPase |
| NT01LR0063 | Microcompartments protein |
| **Hypothetical protein** |  |
| NT01LR0282 | Conserved hypothetical protein |
| NT01LR0849 | Hypothetical protein |
| NT01LR0279 | Conserved hypothetical protein |
| NT01LR1034 | Hypothetical protein |
| NT01LR1682 | Hypothetical protein |
| NT01LR1679 | Conserved hypothetical protein |
| NT01LR0372 | Conserved hypothetical protein |
| NT01LR1798 | Conserved hypothetical protein |
| NT01LR1681 | Hypothetical protein |
| NT01LR1057 | Conserved hypothetical protein |
| NT01LR1688 | Conserved hypothetical protein |
| NT01LR0466 | Hypothetical protein |
| NT01LR1675 | Hypothetical protein |
| NT01LR1033 | Conserved hypothetical protein |
| NT01LR1687 | Conserved hypothetical protein |
| NT01LR1686 | Conserved hypothetical protein |
| NT01LR0610 | Conserved hypothetical protein |
| NT01LR0291 | Hypothetical protein |
| NT01LR0716 | Conserved hypothetical protein |
| NT01LR0450 | Hypothetical protein |
| NT01LR0717 | Hypothetical protein |
| NT01LR0134 | Conserved hypothetical protein |
| NT01LR1799 | Conserved hypothetical protein |
| NT01LR0915 | Hypothetical protein |
| NT01LR0819 | Conserved hypothetical protein |
| NT01LR1011 | Conserved hypothetical protein |
| NT01LR0766 | Conserved hypothetical protein |
| NT01LR1860 | Hypothetical protein |
| NT01LR2002 | Hypothetical protein |
| NT01LR0821 | Conserved hypothetical protein |
| NT01LR0447 | Conserved hypothetical protein |
| NT01LR0429 | Conserved hypothetical protein |
| NT01LR0438 | Conserved hypothetical protein |
| NT01LR0612 | Hypothetical protein |
| NT01LR0195 | Conserved hypothetical protein |
| NT01LR0148 | Hypothetical protein |
| NT01LR0957 | Hypothetical protein |
| NT01LR0132 | Conserved hypothetical protein |
| NT01LR0020 | Conserved hypothetical protein |
| NT01LR0365 | Conserved hypothetical protein |
| NT01LR1169 | Hypothetical protein |
| NT01LR0475 | Hypothetical protein |
| NT01LR1265 | Conserved hypothetical protein |
| **Purines, pyrimidines, nucleotides** |  |
| NT01LR1127 | Phosphoribosylamine – glycine ligase |
| NT01LR1128 | Bifunctional purine biosynthesis protein PurH |
| **Unknown function** |  |
| NT01LR0089 | Housekeeping protease |
| NT01LR0714 | Aldehyde dehydrogenase (NAD) family protein |
| NT01LR1179 | Protein lolS |
| NT01LR1802 | Molybdopterin biosynthesis protein, D chain |
| NT01LR0090 | Hydrolase |
| NT01LR0194 | lr1712 |
| NT01LR1178 | Protein of unknown function |
| NT01LR0447 | Esterase |
| NT01LR0319 | spfh domain |
| **Biosynthesis of cofactors** |  |
| NT01LR1356 | Putative siroheme synthase |
| NT01LR1803 | Molybdopterin converting factor, subunit 2 |
| NT01LR0312 | FeS assembly protein SufB |
| NT01LR1782 | 4-methyl-5-(beta-hydroxyethyl)-thiazole monophosphate biosynthesis enzyme |
| NT01LR1780 | Thiamine-phosphate pyrophosphorylase |
| NT01LR0619 | Amidase |
| NT01LR1329 | Phosphomethylpyrimidine kinase |
| **Mobile and extrachromosomal element functions** |  |
| NT01LR0820 | Prophage superinfection immunity protein |
| NT01LR1859 | Transposase |
| NT01LR0974 | Prophage antirepressor |
| **Regulatory functions** |  |
| NT01LR1856 | Putative transcriptional regulator family |
| NT01LR1809 | Transcriptional regulator, xre family |
| NT01LR0822 | Repressor |
| NT01LR0093 | Sensor protein YycG |
| NT01LR0908 | Transcriptional regulator |
| NT01LR0936 | Transcriptional regulator |
| NT01LR0846 | LexA repressor |
| NT01LR0094 | Response regulator |
| **Signal transduction** |  |
| NT01LR1797 | Response regulator |
| **DNA metabolism** |  |
| NT01LR0253 | Protein in LdhD 5’ region |
| NT01LR0818 | Integrase |
| NT01LR1301 | DNA-3-methyladenine glycosylase 1 |
| NT01LR0518 | ATP-dependent DNA helicase RecQ |
| NT01LR0468 | Type III restriction enzyme, res subunit |
| **tRNA and rRNA base modification** |  |
| NT01LR1963 | tRNA-Arg |
| **Amino acid biosynthesis** |  |
| NT01LR0715 | Pyrroline-5-carboxylate reductase |
| **Protein fate** |  |
| NT01LR0660 | Chaperonin GroL |
| NT01LR1240 | Methionine-S-sulfoxide reductase |
| NT01LR0843 | Dipeptidase A |
