# Supplementary material for: FolC2‐mediated folate metabolism contributes to suppression of inflammation by probiotic Lactobacillus reuteri
Source: Microbiologyopen. 2016 Jun 28;5(5):802–18. doi: 10.1002/mbo3.371 (PMC5061717; doi:10.1002/mbo3.371)
Supplement: Supplementary file 3 — Table S2. Bacterial strains, vectors, and primers used in this study. [file MBO3-5-802-s003.docx]

**Supplemental Table S2. Bacterial strains, vectors and primers used in this study**

| ***L. reuteri* Strains** | **Description** | **Source** | |
| --- | --- | --- | --- |
| ATCC PTA 6475 | Isolate from Finnish mother’s milk | BioGaia AB (Stockholm, Sweden) | |
| 6475::*folC2*  (HMPREF0538_20290) | *L. reuteri* ATCC PTA 6475 *folC2*::pORI28, Em^r^, *folC2* insertion mutant | this study | |
| 6475::*folC*  (HMPREF0538_21738) | *L. reuteri* ATCC PTA 6475 *folC*::pORI28, Em^r^, *folC* insertion mutant | this study | |
| ATCC 55730 | Isolate from Peruvian mother’s milk | BioGaia AB | |
| **Vector** | **Description** | **Source** | |
| pVE6007 | Cm^r^ repA-positive temperature-sensitive derivative of pWV01 | (45) | |
| pORI28 | Em^r^ repA-negative derivative of pWV01 | (47) | |
| pCR^®^2.1 | Cloning vector | Invitrogen, Carlsbad, CA | |
| pJKS100 | *E. coli* – *L. reuteri* shuttle vector containing *L. lactis* P_23_ promoter, Cm^r^ | (42) | |
| **Primers** | **Primer sequence (5’ to 3’)** | **Gene targeted** | |
| LR1322F 5’-BHI | TGACGGATCCTAACCATATTGCAGGGACAAACGG, amplification of pORI28 insert | 6475 *folC2* |  |
| LR1322R 3’-ERI | TGACGAATTCCAGCGGGTAAATGTTCGGTGA, amplification of pORI28 insert | 6475 *folC2* |  |
| LR1511F 5’-BHI | TGACGGATCCTAACAGGAACTAATGGTAAGGGATC, amplification of pORI28 insert | 6475 *folC* |  |
| LR1511R 3’-ERI | TGACGAATTCCGCCTGTTTCCAATGTATTATCA, amplification of pORI28 insert | 6475 *folC* |  |
| **Primer Name** | **Probe Sequence – qRT-PCR** | **Universal Probe Number** |  |
| rpoB-F | cgtgatacttcattacgtgttcct | 77 |  |
| rpoB-R | agtgaagactttaacatcttggatga |  |  |
| hdcA-F | gcactaacgataaccgtcgtc | 1 |  |
| hdcA-R | cacccttattagcacaaacaatga |  |  |
| hdcB-F | tgcaagatcggatatcacaaa | 162 |  |
| hdcB-R | ttgacgaattcaaccttctttaca |  |  |
| hdcP-F | tccctacggataccaagcac | 15 |  |
| hdcP-R | agaggaacgctaagacaccaat |  |  |
| narI-F | attcccacaaacgcaagg | 120 |  |
| narI-R | ccacatctttgggtcattgat |  |  |
